# Clinically relevant quality measures for risk factor control in primary care: a retrospective cohort study

**DOI:** 10.1186/1472-6963-14-306

**Published:** 2014-07-15

**Authors:** Stefan Weiler, Armin Gemperli, Tinh-Hai Collet, Douglas C Bauer, Lukas Zimmerli, Jacques Cornuz, Edouard Battegay, Jean-Michel Gaspoz, Eve A Kerr, Drahomir Aujesky, Nicolas Rodondi

**Affiliations:** 1 Department of General Internal Medicine, University of Bern, Bern, Switzerland; 2 Department of Clinical Research, Clinical Trials Unit, University of Bern, Bern, Switzerland; 3 Department of Health Sciences and Health Policy, University of Lucerne, Lucerne, Switzerland; 4 Swiss Paraplegic Research, Nottwil, Switzerland; 5 Department of Ambulatory Care and Community Medicine, University of Lausanne, Lausanne, Switzerland; 6 Service of Endocrinology, Diabetes, and Metabolism, Lausanne University Hospital, Lausanne, Switzerland; 7 Department of Epidemiology and Biostatistics, University of California San Francisco, San Francisco, CA, USA; 8 Department of Medicine, University of California San Francisco, San Francisco, CA, USA; 9 Division of Internal Medicine, University Hospital of Zurich, Zurich, Switzerland; 10 Medical Outpatient Department/Ambulatory Internal Medicine, University Hospital Basel, Basel, Switzerland; 11 Department of Community Medicine and Primary Care, University Hospitals of Geneva and Faculty of Medicine, Geneva, Switzerland; 12 Veterans Affairs Center for Clinical Management Research, HSR&D Center of Excellence; Department of Internal Medicine, University of Michigan, Ann Arbor, MI, USA

**Keywords:** Clinical inertia, Blood pressure, Quality of care, Pharmacological intervention, Hypertension, Dyslipidemia, Diabetes mellitus, Cohort study

## Abstract

**Background:**

Assessment of the proportion of patients with well controlled cardiovascular risk factors underestimates the proportion of patients receiving high quality of care. Evaluating whether physicians respond appropriately to poor risk factor control gives a different picture of quality of care. We assessed physician response to control cardiovascular risk factors, as well as markers of potential overtreatment in Switzerland, a country with universal healthcare coverage but without systematic quality monitoring, annual report cards on quality of care or financial incentives to improve quality.

**Methods:**

We performed a retrospective cohort study of 1002 randomly selected patients aged 50–80 years from four university primary care settings in Switzerland. For hypertension, dyslipidemia and diabetes mellitus, we first measured proportions in control, then assessed therapy modifications among those in poor control. “Appropriate clinical action” was defined as a therapy modification or return to control without therapy modification within 12 months among patients with baseline poor control. Potential overtreatment of these conditions was defined as intensive treatment among low-risk patients with optimal target values.

**Results:**

20% of patients with hypertension, 41% with dyslipidemia and 36% with diabetes mellitus were in control at baseline. When appropriate clinical action in response to poor control was integrated into measuring quality of care, 52 to 55% had appropriate quality of care. Over 12 months, therapy of 61% of patients with baseline poor control was modified for hypertension, 33% for dyslipidemia, and 85% for diabetes mellitus. Increases in number of drug classes (28-51%) and in drug doses (10-61%) were the most common therapy modifications. Patients with target organ damage and higher baseline values were more likely to have appropriate clinical action. We found low rates of potential overtreatment with 2% for hypertension, 3% for diabetes mellitus and 3-6% for dyslipidemia.

**Conclusions:**

In primary care, evaluating whether physicians respond appropriately to poor risk factor control, in addition to assessing proportions in control, provide a broader view of the quality of care than relying solely on measures of proportions in control. Such measures could be more clinically relevant and acceptable to physicians than simply reporting levels of control.

## Background

Although a broad armamentarium of pharmacotherapeutic interventions and recommendations are available, cardiovascular risk factors are often suboptimally controlled. Clinical inertia in the form of insufficient treatment intensification in the face of poor disease control has been suggested to be a major cause of failure to respond to abnormal measurements [1,2]. While quality of care measures should ideally reflect whether physicians and systems deliver appropriate clinical action, most current measures focus on achievement of a target rather than changes in treatment [3].

We have previously shown the feasibility of measuring physician response to poor risk factor control in the US using electronic treatment records as an additional “tightly linked” clinical action measure of quality [4,5]. Provider responses to poorly controlled risk factor levels such as intensification of pharmacotherapy are tightly linked clinical action measures, which are clinically relevant indicators for quality of care directly linked to improved patient outcomes, further improving quality assessment and reducing risks of overtreatment [3,4,6]. The primary goal of new action measures is the improvement of quality of care. Focusing on accountability measures might corrupt the process of monitoring quality of care [7].

Recently, new measures have also been developed for potential overtreatment of cardiovascular risk factors, such as hypertension and diabetes mellitus [3,8]. However, limited data exist about physician response to poor risk factor control and markers of overtreatment in settings without systematic quality monitoring. In Switzerland, systematic quality monitoring and annual report cards on quality of care [9], and financial incentives to improve quality, are not implemented. Among other differences from the US, all patients have universal healthcare coverage in Switzerland.

In the present study, we assessed physician response to control of hypertension, dyslipidemia, and diabetes mellitus, as well as markers of potential overtreatment, among a random sample of 1002 patients aged 50–80 years followed for two years in four Swiss University primary care settings.

## Methods

### Study participants

We abstracted medical charts from 1002 randomly selected patients from Swiss university primary care settings in Basel, Geneva, Lausanne and Zürich to establish a retrospective cohort study over 2 years, as described previously in detail [10]. The Institutional Review Boards at each site approved the study. This study was approved by the Ethics Committee of Zürich, the Human Research Ethics Committee of Geneva, the Human Research Ethics Committee of Vaud, and the Ethics Committee of Basel, at the sites of Zürich, Geneva, Lausanne, and Basel, respectively. The random sample was drawn from electronic administrative data of all patients aged 50 to 80 years followed in 2005–2006. We limited our sample to this age group to have a high enough prevalence of cardiovascular risk factors. Patients were followed by residents in general internal medicine at the end of their postgraduate training who were supervised by university attendings, or were seen directly by university attendings (10%). Patients who were followed for less than one year, in a specialized clinic only or who had no outpatient visit to a primary care physician were excluded. Nine medical students were centrally trained at one site (Lausanne) for data abstraction from medical charts in each Swiss university primary care setting, and then entered data using EpiData software (version 3.1, EpiData Association, Denmark). We used the same criteria for the identification of patients with hypertension, dyslipidemia, diabetes mellitus as previously published (Additional file 1: Table S1) [5].

### Diagnostic criteria for “Appropriate Clinical Action”

Among patients with poorly controlled hypertension, dyslipidemia, or diabetes mellitus during the study period, we assessed any therapy modification response to one or more poorly controlled conditions [5]. Patients with near control of risk factors were not analyzed for therapy modifications because of the appropriateness of lifestyle modifications for near control of these conditions. Patients with diabetes already treated with insulin therapy were not included into the analysis of therapy modifications, because day-to-day adjustments in insulin dosage could not be reliably identified.

We defined pharmacotherapeutic intervention as an increase in the number of different drug classes, an increase in the dosage of one or more drugs, or a switch to another drug class. We defined “appropriate clinical action” as any of these pharmacotherapeutic interventions or return to control without therapy modification within 12 months as physicians may sometimes opt for non-pharmacological recommendations [5]. Responses were also examined within six months. Other possibilities were considered “inappropriate clinical action” as no return to control, return to “near control”, or no further measurements without any pharmacotherapeutic intervention within 12 months. In terms of quality of care, we compared the differences between simply measuring proportions of patients with controlled risk factors (markers in control) and adding appropriate clinical action for those with poor risk factor control (action measure).

Drugs were grouped into drug classes, with seven antihypertensive classes (thiazides diuretics, other diuretics, beta-blockers, calcium-channel blockers, angiotensin-converting enzyme inhibitors, angiotensin-receptor blockers, other antihypertensives), five lipid-lowering classes (statins, fibric acid derivatives, niacin, bile acid resins, other lipid-lowering agents), and five antidiabetic classes (insulin, sulfonylureas, metformin, thiazolidinediones, other diabetes-related agents). Daily dosages were recorded except for insulin whose day-to-day adjustments could not be reliably identified in this retrospective review of medical charts.

### Comorbidities

We analyzed the associations between various patient factors, such as co-occurrence of several conditions or target organ disease and “appropriate clinical action” for poorly controlled conditions. Target organ disease is based on the definitions of the Joint National Committee 7th report guidelines [11]: any previous diagnosis of hypertensive heart disease, congestive heart failure, cerebrovascular or peripheral arterial disease, and nephropathy. Based on a previous published study [12], another predictor variable was the number of comorbidities. Categories of cardiovascular risk included the history of coronary artery disease (CAD), other target organ disease or no history of either condition. Previous CAD was defined as any diagnosis of myocardial infarction, angina pectoris, atherosclerotic heart disease or coronary revascularization [5].

### Markers of potential overtreatment

Recently, criteria for potential overtreatment of hypertension among patients with diabetes mellitus have been developed [3]. Based on this study and on current guidelines at the time of the patient care [11,13], we defined potential overtreatment of hypertension among diabetic patients if both low systolic (<130 mmHg) and low diastolic (<65 mmHg) values, and receiving three or more antihypertensive drugs applied to diabetics and to the overall sample. We defined potential overtreatment of dyslipidemia as having low LDL cholesterol (<2.6 mmol/L or <100 mg/dL) and a high daily dose of statin (atorvastatin ≥40 mg, rosuvastatin ≥10 mg, simvastatin ≥40 mg) for individuals without a diagnosis of cardiovascular disease (primary prevention) [14]. We developed new criteria for potential overtreatment of diabetes mellitus based on the new ADA guidelines which state that less stringent glycosylated haemoglobin (HbA1c) goals are possibly appropriate for patients with limited life expectancy, extensive comorbid conditions^13^, and supported by a commentary by Pogach and Aron [8]. Potential overtreatment of patients with diabetes was defined as patients with a low HbA1c <7.0%, on treatment with two or more glucose-lowering agents, when the patient had multi-morbidity (defined as two or more diseases, excluding cardiovascular risk factors) [15,16] or a short life expectancy (defined as a terminal illness, such as cancer). We did not have any longitudinal data on therapy intensification among patients with controlled risk factors, as these data were not collected.

### Statistical analysis

For patients with poorly controlled hypertension, dyslipidemia and diabetes mellitus, we examined percentages of pharmacotherapeutic interventions or return to normal levels, as described above. We examined different factors associated with “appropriate clinical action” at six and twelve months using multivariable logistic regression. We used mixed-effects logistic regression (Stata version 12.0, Stata Corp., College Station, TX) to account for clustering by the four sites as a fixed factor and physicians as random factor [17].

## Results

### Population characteristics and degree of control

**Figure 1:**
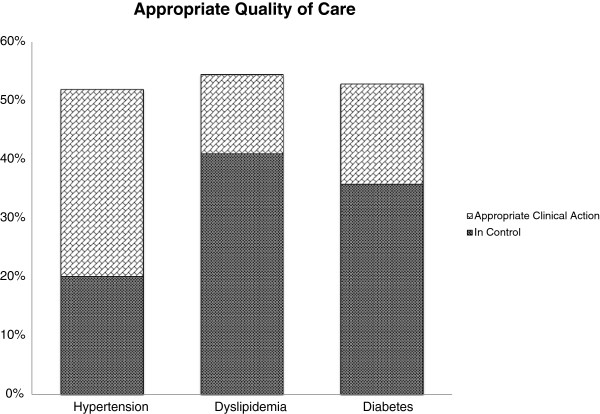
**Quality of care measurements.** Comparison of appropriate quality of care by simple measuring proportions in control (markers in control) and appropriate clinical action for poor risk factor control including physicians’ response (action measure). Patients with baseline risk factors in control: hypertension n = 151/753 (20%), dyslipidemia 264/644 (41%), diabetes 105/293 (36%). Patients with appropriate clinical action within 12 months: hypertension 240/753 (32%), dyslipidemia 87/644 (14%), diabetes 50/293 (17%).

Baseline demographic characteristics and comorbid conditions are described in Table 1. Among the 1002 patients at baseline, 753 had hypertension (20% in control and 52% poorly controlled), 644 had dyslipidemia (41% in control, 36% poorly controlled), and 293 had diabetes (36% in control, 20% poorly controlled) (Figure 1). When appropriate clinical action in response to poor control over a 12 month period was integrated into measuring quality of care, the proportion of patients receiving appropriate/high quality of care increased to 52% of patients with hypertension (391 of 753 patients), 55% of patients with dyslipidemia (351 of 644) and 53% of patients with diabetes mellitus (155 of 293 patients) (Figure 1). The highest number of condition-specific medications was given to patients with poorly controlled hypertension, with 30% receiving three or more anti-hypertensive drugs at baseline. More than 60% of patients with poorly controlled hypertension were on three or more medications at baseline. Patients with poorly controlled dyslipidemia received the lowest number of condition-specific drugs at baseline and 71% were untreated at baseline. One to two thirds of patients with one poorly controlled condition had also the other two conditions (Table 2).

**Table 1:** Baseline characteristics of patients with poorly controlled hypertension, dyslipidemia, and diabetes mellitus

	**Poorly controlled Hypertension (n = 391)**		**Poorly controlled Dyslipidemia (n = 231)**		**Poorly controlled diabetes Mellitus (n = 59)**	
**Demographic**						
Age, years Mean, (SD)	64.5	(8.1)	62.9	(7.7)	62.8	(8.6)
	**n**	**%**	**n**	**%**	**n**	**%**
Age						
< 65 years	193	49	130	56	32	54
≥ 65 years	198	51	101	44	27	46
Sex						
Female	159	41	99	43	22	37
**Baseline medications**						
**Medication for each condition**						
0 medications	67	17	165	71	15	25
1 medications	92	24	64	28	18	31
2 medications	115	29	2	1	25	42
3 medications	74	19	0	0	1	2
≥4 medications	43	11	0	0	0	0
**Medication for others of the 3 conditions**						
0 medications	109	28	60	26	9	15
1 medications	98	25	37	16	14	24
2 medications	85	22	52	23	10	17
3 medications	46	12	30	13	13	22
≥4 medications	53	14	52	23	13	22
**All medications**						
0 medications	17	4	22	10	3	5
1 medications	42	11	34	15	6	10
2 medications	52	13	36	16	3	5
3 medications	57	15	42	18	12	20
≥4 medications	223	57	97	42	35	59
**Citizenship**						
Switzerland	171	44	97	42	22	37
Europe/USA	80	20	48	21	10	17
Eastern Europe	83	21	46	20	19	32
Africa	26	7	18	8	5	8
Latin America	15	4	9	4	2	3
Asia/Middle-East	14	4	13	6	1	2
**Civil status**						
Single	51	13	34	15	3	5
Divorced/separated	87	22	63	27	17	29
Widowed	46	12	22	10	6	10
Married	203	52	111	48	33	56
**Occupation**						
Social aid	46	12	25	11	7	12
Unemployed	40	10	20	9	8	14
Employed	92	24	73	32	10	17
Retired	159	41	86	37	26	44
At home	45	12	25	11	7	12
Other	2	1	0	0	1	2

Missing data on citizenship for 2 patients with poorly controlled hypertension, on civil status for 4 patients with poorly controlled hypertension and 1 patient with poorly controlled dyslipidemia, on occupation for 7 patients with poorly controlled hypertension, for 2 with poorly controlled dyslipidemia, respectively.

**Table 2:** Comorbid conditions of the patients with poorly controlled conditions

**Co-Conditions and Co-Morbidities**						
	**Hypertension poor control (n = 391)**		**Dyslipidemia poor control (n = 231)**		**Diabetes mellitus poor control (n = 59)**	
	**n**	**%**	**n**	**%**	**n**	**%**
**Conditions**						
all three conditions	141	36	60	26	41	69
+ hypertension			187	81	51	86
+ dyslipidemia	294	75			48	81
+ diabetes mellitus	169	43	70	30		
**Comorbidities**						
Cerebrovascular Disease (TIA, CVA)	56	14	20	9	6	10
Coronary Artery Disease	75	19	37	16	9	15
Congestive Heart Failure	28	7	11	5	3	5
End Stage Renal Disease	2	1	1	0	0	0
Dementia	9	2	2	1	1	2
COPD	50	13	28	12	10	17
Asthma	16	4	6	3	3	5
Gastric Ulcer	13	3	12	5	5	8
Breast Cancer	6	2	4	2	0	0
Colorectal Cancer	8	2	0	0	1	2
Prostate Cancer	16	4	8	3	2	3
Depression	68	17	46	20	12	20
**Number of Comorbidities**						
Mean, (SD)	2.3 (1.0)		1.9 (1.1)		2.7 (1.0)	
Distribution						
0	0	0	17	7	0	0
1	87	22	72	31	3	5
2	158	40	81	35	25	42
>2	146	37	61	26	31	53

COPD chronic obstructive pulmonary disease.

### Therapy modifications during 6-month and 12-month periods

Within six months, 49% of patients received therapy modification for poorly controlled blood pressure, 24% for poorly controlled LDL cholesterol level, and 75% for poorly controlled HbA1c (Table 3). In most patients therapy was modified by adding another drug class (19-44%) or by increasing the dosage (7-53%). Within a longer observation period of 12 months, patients with therapy modifications slightly increased to 61% for poorly controlled blood pressure, 33% for poorly controlled LDL-cholesterol level, and 85% for poorly controlled HbA1c. Although the number of patients with therapy modifications rose between 6 and 12 months, a majority of patients had drug modifications within the first six months. Only a small number of patients (0-5%) returned to control after 12 months without any therapy modifications.

**Table 3:** Patients with poorly controlled hypertension, dyslipidemia, or diabetes mellitus, who had subsequent therapy modifications within a 6-months or 12 months-period

	**Patients with poorly controlled hypertension**				**Patients with poorly controlled dyslipidemia**				**Patients with poorly controlled diabetes mellitus**			
	**6 Months**		**12 Months**		**6 Months**		**12 Months**		**6 Months**		**12 Months**	
	**n**	**%**	**n**	**%**	**n**	**%**	**n**	**%**	**n**	**%**	**n**	**%**
**Appropriate clinical action**	211	54.0	240	61.4	67	29.0	87	37.7	46	78.0	50	84.7
Any therapy modification	193	49.4	238	60.9	55	23.8	77	33.3	44	74.6	50	84.7
Increase class	121	30.9	153	39.1	43	18.6	64	27.7	26	44.1	30	50.8
Increase dose	103	26.3	135	34.5	15	6.5	23	10.0	31	52.5	36	61.0
Switch class^†^	30	7.7	49	12.5	0	0.0	2	0.9	6	10.2	10	16.9
Several modifications	55	14.1	88	22.5	3	1.3	11	4.8	19	32.2	26	44.1
Return to control WO modification^‡^	18	4.6	2	0.5	12	5.2	10	4.3	2	3.4	0	0.0
**Inappropriate clinical action**	180	46.0	151	38.6	164	71.0	144	62.3	13	22.0	9	15.3
No modification, return to near control^‡^	25	6.4	9	2.3	38	16.5	27	11.7	1	1.7	3	5.1
No modification, no return^‡^	120	30.7	134	34.3	40	17.3	40	17.3	7	11.9	2	3.4
No measurement, no modification	35	9.0	8	2.0	86	37.2	77	33.3	5	8.5	4	6.8

LDL = low-density lipoprotein. ^†^A switch to different drug class was counted when a new class of medication for the condition was added, but the total number of drug classes remained the same. ^‡^Categories of control with levels of control, near and poor control for hypertension, dyslipidemia and diabetes mellitus are based on N. Rodondi et al. [5]. This category includes patients without visit or measurements.

About one third of hypertensive patients with inappropriate clinical action remained in poor control without therapy modification. In contrast, more than half of the patients receiving inappropriate clinical action for poorly controlled dyslipidemia had no further LDL-cholesterol measurement within 6 or 12 months. In patients with poorly controlled diabetes mellitus and inappropriate clinical action about 70% were already receiving one or two oral antidiabetics (77% after six months, 67% after 12 months). In contrast, among patients with poorly controlled dyslipidemia and inappropriate clinical action about 70% were not on any single lipid-lowering medication at baseline.

### Multivariable analysis of factors associated with appropriate clinical action

At 12 months, previous target organ disease and CAD were independently associated with appropriate clinical action in response to poor control of dyslipidemia (Table 4). Higher baseline levels of systolic and diastolic blood pressure and LDL cholesterol levels were associated with a higher proportion of appropriate clinical action for poorly controlled hypertension and dyslipidemia. Patient gender or age did not influence appropriate clinical action. Hypertensive patients with CAD or target organ damage were not more likely to receive appropriate clinical action in response to elevated blood pressure, than those without these conditions using multivariable analyses.

**Table 4:** Multivariable analysis of factors associated with “Appropriate Clinical Action” in response to poorly controlled hypertension, dyslipidemia, and diabetes mellitus at 12 months

	**Poorly controlled hypertension (n = 391)**			**Poorly controlled dyslipidemia (n = 231)**			**Poorly controlled diabetes Mellitus (n = 59)**		
	**Proportion**	**OR**	**95% CI**	**Proportion**	**OR**	**95% CI**	**Proportion**	**OR**	**95%CI**
	**(%)**			**(%)**			**(%)**		
**Age**									
<65 years	59.0			35.8			98.2		
≥65 years	66.6	1.38	(0.86-2.22)	31.9	0.84	(0.41-1.71)	97.9	0.86	(0.14-5.23)
**Sex**									
Female	64.5			40.5			98.5		
Male	61.8	0.89	(0.56-1.43)	29.7	0.62	(0.29-1.30)	97.6	0.60	(0.09-3.99)
**Co-occurrence of other conditions**									
no co-occurrence of HT, DL, DM	62.0			42.0			NA		
HT and DL	67.1	1.25	(0.64-2.41)	30.8	0.62	(0.19-1.98)			
HT and DM	62.8	1.03	(0.35-3.06)				76.3	ref	
DL and DM				9.4	0.14	(0.01-1.43)	100.0	NA	
All 3 conditions	58.7	0.87	(0.41-1.86)	43.6	1.07	(0.23-5.00)	90.1	2.81	(0.27-29.11)
**Number of other conditions**									
increase 1 co-morbidity		1.23	(0.93-1.63)		0.92	(0.57-1.49)		0.63	(0.19-2.09)
**Risk status**									
No target organ disease	61.4			17.9			NA		
Target organ disease (except CAD)	65.4	1.19	(0.56-2.52)	49.0	4.41	(1.59-12.19)**	97.6	ref	
CAD	56.0	0.80	(0.31-2.07)	51.0	4.78	(1.27-18.02)*	99.3	3.27	(0.12-87.75)
**Baseline level of each condition**									
Systolic BP									
140-159 mmHg	59.9								
160-179 mmHg	62.6	1.12	(0.67-1.88)						
≥180 mmHg	93.0	8.86	(1.06-73.98)*						
Diastolic BP									
90-99 mmHg	59.9								
100-109 mmHg	73.7	1.88	(0.87-4.08)						
≥110 mmHg	91.0	6.78	(0.71-65.14)						
LDL cholesterol level									
3.4-4.1 mmol/L (130–159 mg/dL)				22.5					
4.2-4.9 mmol/L (160–189 mg/dL)				44.6	2.77	(1.15-6.68)*			
≥5.0 mmol/L (≥190 mg/dL)				54.2	4.08	(1.32-12.62)*			
Haemoglobin A1c									
8.0-8.9%							98.1		
9.0-9.9%							99.0	2.75	(0.12-31.21)
≥10.0%							96.0	0.45	(0.07-3.07)

Hierarchical logistic regression with adjustment for all the covariates in the table. Patient factors associated with “appropriate clinical action” vs. “inappropriate clinical action” at 12 months were assessed in these multivariable models. “Appropriate clinical action” was defined as therapy modifications or return to control without therapy modification. BP = blood pressure; CAD = coronary artery disease; LDL = low-density lipoprotein; HT = hypertension; DL = dyslipidemia; DM = diabetes mellitus. OR = odds ratio; 95% CI = 95% confidence interval; NA = not available. Odds Ratios are related to the first class shown for each category. Due to low number of patients with poorly controlled diabetes mellitus some calculations for proportions, odds ratios and 95% confidence interval are not feasible. Ref = reference category in diabetes mellitus due to low number of patients. *P < 0.05 compared to reference group; **P < 0.01 compared to reference group.

### Potential overtreatment of hypertension, dyslipidemia and diabetes mellitus

Five out of 256 (2%) diabetic patients with hypertension met the criteria for overtreatment. Overall, we found 2% of patients (15/753) to be potentially overtreated for hypertension, 3% (8/293) for diabetes mellitus, and 3% for dyslipidemia (19/644) whereas 6% of patients with diabetes had potential overtreatment for dyslipidemia (13/236).

## Discussion

In Switzerland, a country without systematic quality monitoring, we found that measuring proportions in control without evaluating whether physicians respond appropriately to poor risk factor control largely underestimated quality of care. When appropriate clinical action in response to poor control over a 12 month period was integrated into measuring quality of care compared to the measurement of proportions of patients with controlled risk factors, proportions with appropriate quality of care increased for hypertension (20% vs. 52%), for dyslipidemia (41% vs. 55%), and for diabetes mellitus (36% vs. 53%).

Suboptimal care for poorly controlled cardiovascular risk factors was provided to 15% to 62% of patients at 12 months in our study. Failure to respond to poorly controlled measurements is a barrier to good clinical and appropriate clinical action of cardiovascular risk factors [1]. Guidelines are frequently changing. For example, recent evidence suggests that treatment with moderate dose statins is more important than achieving target values for LDL [14]. However, at baseline over 35% of patients at high risk of cardiovascular events with an LDL >2.6 mmol/L were not treated with statins in our study. Other reasons for inappropriate clinical action might be the patients’ lack of adherence to pharmacological therapy, missed appointments, missed laboratory tests or the recommendation for lifestyle modifications [18], which could not directly be identified in our data. However, return to control without any treatment, possibly due to lifestyle modifications, was counted as appropriate clinical action in our study.

To our knowledge, this is the first study in a country without systematic quality monitoring, to document appropriate physician response to poor control of cardiovascular risk factors and potential overtreatment. Systematic quality monitoring is widely used in the US and in the UK [16]. It has been shown that higher quality of care might be delivered when performance measures and monitoring are established [19]. Other factors influencing physicians’ prescription behaviour are likely to be similar to those in the US setting, such as adherence to the same US treatment guidelines (or slight adaptation of them) and potential industrial influence, with several industrial headquarters in Switzerland. As most physicians in our study had a fixed salary, it is unlikely that financial incentives played a significant role in our results. Previous studies on quality of care based on therapy modifications in response to cardiovascular risk factors were primarily performed in countries with quality monitoring such as the US [5,7,20-22]. In a previous study from the US, the rates of therapy modifications for poorly controlled blood pressure were comparable to our study, slightly lower for poorly controlled HbA1c, and higher for poorly controlled LDL cholesterol levels [7]. In contrast, recent studies from the US Department of Veterans Affairs showed higher levels of therapy modification and treatment, along with higher levels of potential overtreatment. In these studies, Kerr and colleagues showed that high rates of performance on measures that assessed attainment of risk factor thresholds may be associated with overtreatment [3,14], while our study found high levels of therapy modification with low rates of overtreatment.

In the US studies previously reported, patients with 1 or more of the 3 conditions, those with higher baseline values of cardiovascular risk factors, target organ damage, younger age or more “routine visits” were more likely to receive appropriate clinical action for all of the three risk factors [5,23-25].

Higher rates of appropriate clinical action for poorly controlled hypertension and dyslipidemia were found among patients with higher baseline levels of blood pressure and LDL cholesterol levels, respectively. Potential overtreatment of these conditions seemed to be low for patients with already well controlled risk factors.

Results from our study show, that high rates of appropriate clinical action and low proportions of overtreatment may also be achieved in a country without systematic quality monitoring. In Switzerland all patients have universal healthcare coverage, including adults with low income who receive social aid to cover healthcare costs, regardless of their age or whether they work. Meeting clinical action measures rather than the treat-to-target approach of surrogate markers is now more and more recommended to improve appropriateness of care [3,8,14]. These measures include clinical processes that are associated with important outcomes. Simply assessing measures of cardiovascular risk factor control without action measures largely minimizes the quality of care provided by physicians and other healthcare providers. We suggest that assessment of quality of care can be improved by including measures of therapy intensification and physician action in the face of uncontrolled values.

Overtreatment might reflect overaggressive and potentially dangerous lowering of markers among low-risk patients, and the use of expensive condition-specific drugs with unproven mortality and/or morbidity benefits [26]. Overtreatment of cardiovascular risk factors seemed to be low in the present cohort. In a recent study, Kerr et al. found 8% of potential overtreatment for hypertension among patients with diabetes mellitus with already low blood pressure (<130/65 mmHg) [3]. In the present study, we extended this analysis to hypertensive patients without diabetes mellitus, and found the same rate of potential overtreatment of hypertension in the two groups (2%). Overtreatment of hyperlipidemia has been shown to be common in the outpatient setting (8-13%) [14,27,28].

Our study has several limitations. First, we could not identify the many potential causes of nonresponse to poor control and the reasons for intensified therapy in the face of low surrogate markers. Second, for overtreatment, widely used guidelines at the time of data collection, such as the American Diabetes Association (ADA) or Seventh Report of the Joint National Committee on Prevention, Detection, Evaluation, and Treatment of High Blood Pressure (JNC 7) [11,13] do not specify lower thresholds of blood pressure or HbA1c goals. Recent large studies have not shown significant mortality benefit among patients intensively treated for hypertension and diabetes mellitus [29-31], while one group reported increased mortality after intensive glucose lowering therapy in type 2 diabetes [32]. The ADA now mentions that less stringent treatment goals (HbA1c > 7%) may be appropriate for adults with limited life expectancy or co-morbidities [13]. The definitions of overtreatment are recent and no consensus on these new markers have been reached. To define overtreatment, we used recent publications and widely used guidelines. Third, data from the present cohort were obtained in university settings and results might differ for general practitioners in the community. Care provided by university attendings and residents might be more guideline driven, leading to increased percentage of appropriate clinical action, but also potential overtreatment. Fourth, for diabetes mellitus, our measure of physician response was not applicable to patients with insulin treatment as data on day-to-day adjustments of dosages were not reliably recorded. While we have previously shown that treatment intensification was tightly linked to improved risk factor control [4], our study did not allow conclusions on morbidity or mortality of cardiovascular risk factors with regard to quality of care including action measures. Lastly, we could not assess the predictors of each component of appropriate clinical action, because the sample size was too small for multivariate analyses.

## Conclusions

In summary, evaluating whether physicians respond appropriately to poor risk factor control, in addition to proportions of patients with controlled risk factors, might provide a more clinically relevant index of quality of care. This might result in a broader view of the quality of care than relying solely on measures of proportions in control. Such measures could be more clinically relevant and acceptable to physicians than simply reporting levels of control. Overall, rates of overtreatment for cardiovascular risk factors were low, ranging from 2% to 6%. Sophisticated quality measures including action measures may provide a better picture of the quality of care than relying only on measures of proportions in control.

## Competing interests

The authors declare that they have no competing interests.

## Authors’ contributions

SW and NR wrote the manuscript, NR designed the research, LZ, JC, EB, JMG, NR performed the research in the different Universities, SW, AG and THC performed the statistical analyses, DB, EAK and DA contributed new analytical tools. All authors’ read and approved the final manuscript.

## Pre-publication history

The pre-publication history for this paper can be accessed here:

http://www.biomedcentral.com/1472-6963/14/306/prepub

## Supplementary Material

Additional file 1: Table S1 Diagnostic Criteria for Diabetes Mellitus, Hypertension, and Dyslipidemia; adapted from reference [5]. Click here for file
