# Supplementary material for: Clinically relevant quality measures for risk factor control in primary care: a retrospective cohort study
Source: BMC Health Serv Res. 2014 Jul 15;14:306. doi: 10.1186/1472-6963-14-306 (PMC4105792; doi:10.1186/1472-6963-14-306)
Supplement: Additional file 1: Table S1 — Diagnostic Criteria for Diabetes Mellitus, Hypertension, and Dyslipidemia; adapted from reference [5]. [file 1472-6963-14-306-S1.docx]

Additional file

Table 1

Diagnostic Criteria for Diabetes Mellitus, Hypertension, and Dyslipidemia; adapted from reference [5].

Diabetes Mellitus (one of the following):

| 1) | at least 1 prescription of insulin or an oral hypoglycemic agent; or |
| --- | --- |
| 2) | at least 2 outpatient diagnoses of diabetes mellitus; or |
| 3) | one outpatient diagnosis of diabetes mellitus plus at least 1 measurement of HbA1c ≥ 7%; or |
| 4) | at least 1 hospital discharge with a primary DM-related diagnosis (ICD-9 code 250.X). |

Hypertension (one of the following):

| 1) | at least 1 prescription for an anti-hypertensive medication plus an outpatient diagnosis of hypertension; or |
| --- | --- |
| 2) | at least 2 outpatient diagnoses of hypertension; or |
| 3) | at least 1 prescription for an anti-hypertensive medication plus 1 or more elevated outpatient blood pressure readings (≥140 mmHg systolic, or ≥ 90 mmHg diastolic); or |
| 4) | at least 1 outpatient diagnosis of hypertension plus at least 1 blood pressure reading of ≥ 140 mmHg systolic or ≥ 90 mmHg diastolic; |

Dyslipidemia (one of the following):

| 1) | at least 1 prescription for a lipid-lowering agent; or |
| --- | --- |
| 2) | Outpatient diagnosis of hyperlipidemia/hypercholesterolemia with a prior LDL-cholesterol value ≥ risk-appropriate cutpoint value, as defined in Ref. 5 under “in control”. |

ICD-9 = International Classification of Diseases, Ninth Revision; LDL = low-density lipoprotein; Hb A1C = haemoglobin A1C.
